# STRENGTHENING CAREGIVING NETWORKS: PROMISING PRACTICES TO SUPPORT PAID AND FAMILY CARE TEAMS IN THE HOME

**DOI:** 10.1093/geroni/igad104.1520

**Published:** 2023-12-21

**Authors:** Emily Franzosa, Katherine Ornstein

## Abstract

Older adults and people with disabilities often rely on both family and paid caregivers to live independently in the community. But while paid and family caregivers work closely together in the home, these groups are often siloed in research and policy. Research describing the structure and work of caregiving teams is limited, as are efforts to better support them in providing person-centered care. In this symposium, we present innovative interdisciplinary research that highlights the vital relationship between paid and family caregivers and how this influences the care they provide. We begin with two studies focusing on the structure and function of older adults’ formal and informal care networks. First, Franzosa et al present results from a care mapping project which found care networks were often far more expansive than what is captured in medical records. Reckrey et al describe the need to provide family-centered home care that reflects the nuanced and individualized care that these teams provide, based on a study of interviews with families and paid aides. Next, we describe results of two promising interventions to support family and paid care in the home. Weidenbacher et al describe a community health worker-led navigation program connecting family caregivers of seriously ill veterans to supportive resources, and Scales and King describe a training for home care workers and family members to facilitate collaborative problem solving and person-centered care. Taken together, these studies broaden our understanding of care teams in the home and highlight promising opportunities to support team-based home care. This is a collaborative symposium between the Family Caregiving and Paid Caregiving Interest Groups.

